# Severe Community-Acquired Bloodstream Infection with *Acinetobacter ursingii* in Person who Injects Drugs

**DOI:** 10.3201/eid2201.151298

**Published:** 2016-01

**Authors:** Helmut J.F. Salzer, Thierry Rolling, Stefan Schmiedel, Eva-Maria Klupp, Christoph Lange, Harald Seifert

**Affiliations:** University Medical Center Hamburg–Eppendorf, Hamburg, Germany (H.J.F. Salzer, T. Rolling, S. Schmiedel, E.-M. Klupp);; Research Center Borstel, Sülfeld, Germany (H.J.F. Salzer, C. Lange);; Karolinska Institute, Stockholm, Sweden (C. Lange);; University of Namibia School of Medicine, Windhoek, Namibia (C. Lange);; German Center for Infection Research, Braunschweig, Germany (C. Lange, H. Seifert);; University of Cologne, Cologne, Germany (H. Seifert)

**Keywords:** Acinetobacter ursingii, bacteria, community-acquired bloodstream infection, severe sepsis, person who injects drugs, matrix-assisted laser desorption/ionization time-of-flight mass spectrometry, MALDI-TOF

## Abstract

We report a community-acquired bloodstream infection with *Acinteobacter ursingii* in an HIV-negative woman who injected drugs. The infection was successfully treated with meropenem. Species identification was performed by using matrix-assisted laser desorption/ionization time-of-flight mass spectrometry. Improved identification of *Acinetobacter* spp. by using this method will help identify clinical effects of this underdiagnosed pathogen.

*Actinetobacter* spp. are a diverse group of gram-negative, aerobic coccobacilli. Currently, >50 *Acinetobacter* spp. are recognized; most are not pathogenic to humans. Most human infections are nosocomial and predominantly caused by *A. baumannii* and other members of the *A. baumannii* group, including *A. nosocomialis*, *A. pittii*, and *A. seifertii* (*1*). Novel species, such as *A. ursingii* and *A. schindleri*, which have the potential to cause severe opportunistic infections, are emerging (*2*–*8*). Community-acquired *Acinetobacter* spp. infections are reported mainly from tropical regions. Most patients infected have community-acquired pneumonia caused by *A. baumannii* (*1*). We report a case of community-acquired bloodstream infection with *A*. *ursingii* in an HIV-negative woman who injected drugs.

## The Case-Patient

In March 2014, a 47-year-old woman was hospitalized at the Bernhard Nocht Clinic (Hamburg, Germany) with chills, nausea, and fever (temperature <40°C). The patient had a history of intravenous drug use (cocaine and benzodiazepines) and was currently enrolled in a methadone substitution program. She also had chronic hepatitis C virus infection that was successfully treated with pegylated interferon-α, ribavirin, and boceprevir. The patient had a sustained virologic response and negative results for hepatitis C virus RNA at follow-up without progression to liver fibrosis or cirrhosis.

At admission, her peripheral blood leukocyte count was 6.6 × 10^3^ cells/μL, and her C-reactive protein level was increased (29 mg/L [reference value <5 mg/L]). Other routine serum and urine chemical test results were unremarkable. HIV-1/2 infection was not detected. Chest radiograph showed no abnormalities. Soft tissue abscess was not observed, but the patient admitted having injected cocaine and unknown crushed tablets intravenously the day before admission. Toxicologic screening showed highly increased levels of benzodiazepine (>5,000 ng/mL) and cocaine (>1,000 ng/mL) (reference values <20 ng/mL for both substances). Three blood cultures were collected before empiric antimicrobial therapy with intravenous ceftriaxone (2 g 1×/d) was given on day 1.

The next day, the patient’s condition deteriorated. Her leukocyte count increased to 27.6 ×10^3^ cells/μL, and C-reactive protein level increased to 112 mg/L. She was highly febrile and became hypotensive and tachycardic, which are compatible with severe sepsis. The patient responded rapidly to fluid substitution, became hemodynamically stable, and did not need vasopressors. Antimicrobial therapy was changed empirically to meropenem (1,000 mg 3×/d) on day 2.

After 18 h of culture incubation, microbial growth was detected in 3 aerobic blood culture bottles inoculated at admission. Gram staining showed gram-negative rods, and matrix-assisted laser desorption ionization/time-of-flight mass spectrometry fingerprinting with direct sample deposition without extraction (Bruker Daltonim GmbH, Bremen, Germany) identified *A*. *ursingii* (score 2.19). A BLAST search (http://www.ncbi.nlm.nih.gov/BLAST) of partial 16S rRNA gene sequence was performed by using a taxonomy browser (http://www.ncbi.nlm.nih.gov) and showed 99% identity with the *A. ursingii* type strain NBRC110605 (GenBank accession no. LC014147.1).

Antimicrobial susceptibility testing was performed by using Vitek 2 (bioMérieux, Nürtingen, Germany). Results showed susceptibility to imipenem (MIC <0.25 mg/mL), meropenem (<0.25 mg/mL), gentamicin (<1 mg/mL), and ciprofloxacin (<0.25 mg/mL) and resistance to ceftriaxone (32 mg/mL).

The patient became afebrile the next day, her clinical symptoms improved, and laboratory parameters of inflammation returned to reference values. Follow-up cultures remained negative. A transesophageal echocardiogram was unremarkable, without any evidence of infective endocarditis. Antimicrobial drug therapy was continued for 10 days before discharge. At follow-up 2 months later, the patient had no symptoms and was attending group-counseling sessions to maintain drug abstinence.

## Conclusions

After the first description of *A. ursingii* as a new species (*2*), for which 13 of 29 *A. ursingii* isolates were obtained from patients with nosocomial bloodstream infections, few cases of *A. ursingii* bloodstream infections have been reported (Table). We were not able to identify cases of community-acquired *A. ursingii* bloodstream infections in the literature. Loubinoux et al. reported a case of *A. ursingii* bloodstream infection in a severely immunocompromised patient with pulmonary adenocarcinoma who received chemotherapy and corticosteroids (*3*). In that study, *A. ursingii* was associated with an implanted port device and considered of low pathogenic potential.

**Table:** Characteristics of 16 patients with *Acinetobacter ursingii* bloodstream infections*

Patient, age†/sex	Immune status	Clinical manifestation	Acquisition	Treatment	Outcome	Reference
63 y/M	Compromised	Catheter-related bactemia	Nosocomial	Imipenem, amikacin, rifampin	Survived	(*3*)
99 y/M	Competent	Bacteremia, cholangitis	Nosocomial	NR	NR	(*8*)
67 y/F	Competent	Bacteremia, septic shock	Nosocomial	NR	NR	(*8*)
38 y/F	Compromised	Bacteremia	Nosocomial	NR	NR	(*8*)
38 wk/M (newborn)	NR	Wet lung, bacteremia	Nosocomial	Meropenem, amikacin	Survived	(*5*)
23 wk/M (newborn)	NR	Premature, ARDS, bacteremia	Nosocomial	Meropenem, amikacin	Died	(*5*)
37 wk/F (newborn)	NR	Hypoxic ischemic encephalopathy, bacteremia	Nosocomial	Meropenem, amikacin	Survived	(*5*)
32 wk/F (newborn)	NR	Premature, ARDS, bacteremia	Nosocomial	Meropenem, amikacin	Survived	(*5*)
25 wk/M (newborn)	NR	Premature, ARDS, intracranial hemorrhage, bacteremia	Nosocomial	Meropenem, amikacin	Died	(*5*)
36 wk/M (newborn)	NR	Premature, necrotizing enterocolitis, bacteremia	Nosocomial	Ceftazidime, amikacin, immunoglobulin	Survived	(*6*)
29 wk/F (newborn)	NR	Premature, necrotizing enterocolitis, severe hypoxia, bacteremia	Nosocomial	Ciprofloxacin, piperacillin	Died	(*6*)
31 wk/F (newborn)	NR	Premature, ARDS, necrotizing enterocolitis, bacteremia	Nosocomial	Netilmicin, clindamycin;‡ teicoplanin, cefotaxim, clindamycin;§ tobramycin, ciprofloxacin;¶ piperacillin/ tazobactam, vancomycin#	Survived	(*6*)
26/F**	Competent	Bacteremia	Nosocomial	Piperacillin	Survived	(*4*)
30/F**	Competent	Bacteremia	Nosocomial	Cefepime	Survived	(*4*)
NR/NR	Compromised	Bacteremia	Nosocomial	Ciprofloxacin††	Survived	(*9*)
47/F	Competent	Bacteremia, severe sepsis	Community	Meropenem	Survived	This study

*NR, not reported; ARDS, acute respiratory distress syndrome. †Age for the 8 newborns is gestational age. ‡First treatment. §Second treatment. ¶Third treatment. #Fourth treatment. **These women were pregnant. ††Infected with a carbapenem-resistant strain.

However, in the past few years, there have been reports of serious *A. ursingii* bloodstream infections in immunocompromised and immunocompetent patients. Horii et al. reported nosocomial *A. ursingii* bloodstream infections in 2 pregnant women who had no concurrent illnesses (*4*), but the route of transmission was not elucidated. The authors hypothesized that infections might have been acquired from intravenous catheters from the ward shower bath, where *A. ursingii* and 2 different strains of *A. junii* were found. *A. ursingii* was also found associated with nosocomial bloodstream infections among newborns, as demonstrated by 2 outbreaks in neonatal intensive care units that had high mortality rates. In both outbreaks, the source of infection could not be identified. In outbreaks reported by Kilic et al. (*5*) and Máder et al. (*6*), catheter-related infections were assumed. Kilic et al. (*5*) identified the organisms as *A. septicus* sp. nov., which were later shown by Nemec et al. (*7*) to be identical to *A. ursingii*. Dortet et al. (*8*) reported 10 *A. ursingii* infections, which included 3 cases of nosocomial bloodstream infections, but they did not provide clinical details.

Although severe infections with *A. ursingii* are rare, identification of *A. ursingii* by molecular methods, such as 16S rRNA gene sequencing, *rpoB* gene sequence cluster analysis, and amplified fragment length polymorphism fingerprinting, indicates a relatively high prevalence of *A. ursingii* (range 2.6%–4.5%) among clinical *Acinetobacter* spp. isolates (*10*–*13*). In a study from the United Kingdom, *A. ursingii* was identified in 28 (4%) of 690 clinical *Acinetobacter* spp. isolates collected over a 20-month period during 2008–2009; a total of 17 (71%) of 24 *A. ursingii* isolates were recovered from blood cultures, but clinical details were not reported (*12*). Karah et al. (*13*) reported 113 case-patients with *Acinetobacter* spp. bloodstream infections in Norway; 3 (2.6%) were with *A. ursingii*, but clinical details of these patients were not provided. This relatively high incidence is consistent with results from the Netherlands (*10*) and Northern Ireland (*11*).

In most reports, *A. ursingii* isolates were more susceptible to antimicrobial drugs than *A. baumannii* isolates (*4*,*6*,*8*,*12*,*13*). However, Endo et al. (*9*) reported an IMP-1–producing, carbapenem-resistant *A. ursingii* isolate from a patient in Japan with a nosocomial bloodstream infection.

*A. ursingii* isolates are usually resistant to cephalosporins (*3*,*5*,*8*), as was the isolate from the patient we report, which explains the progression from severe sepsis when she was empirically given ceftriaxone to rapid clinical improvement after administration of meropenem. Máder et al. (*6*) reported a premature infant with an *A. ursingii* bloodstream infection also given a cephalosporin (ceftazidime). However, the infant received a combination with amikacin and immunoglobulins for 3 weeks. The infant survived and was discharged.

These findings suggest that *A. ursingii* is an emerging and serious clinical pathogen that is often involved in nosocomial bloodstream infections and associated with use of intravascular catheters. Its natural habitat remains unclear, but so far, it has only been isolated from human clinical sources. The frequency and clinical role of *A. ursingii* might have been underestimated because species identification by phenotypic and semiautomated identification methods is unreliable. For example, Vitek 2 has misidentified *A. ursingii* as *Bordetella bronchiseptica* (*6*,*8*). The most recent software version for Vitek 2 enabled unambiguous identification of *A. ursingii* (H. Seifert, pers. comm.).

We report a case of community-acquired bloodstream infection and severe sepsis caused by *A. ursingii* that was probably associated with intravenous drug use in an immunocompetent patient. This report also highlights the usefulness of matrix-assisted laser desorption/ionization time-of-flight mass spectrometry for identification of *Acinetobacter* spp. We believe that this technology might improve correct species identification of *Acinetobacter* spp., including *A. ursingii*, in routine clinical practice and help elucidate the prevalence and clinical role of this emerging pathogen.
